# Orai3 Calcium Channel Regulates Breast Cancer Cell Migration through Calcium-Dependent and -Independent Mechanisms

**DOI:** 10.3390/cells10123487

**Published:** 2021-12-10

**Authors:** Mohamed Chamlali, Sana Kouba, Lise Rodat-Despoix, Luca Matteo Todesca, Zoltán Pethö, Albrecht Schwab, Halima Ouadid-Ahidouch

**Affiliations:** 1 Laboratory of Cellular and Molecular Physiology, UR UPJV 4667, University of Picardie Jules Verne, 33 Rue Saint Leu, 80000 Amiens, France; mohamedchamlali80@hotmail.fr (M.C.); sana.kouba@u-picardie.fr (S.K.); lise.despoix@u-picardie.fr (L.R.-D.); 2 Institute of Physiology II, University of Münster, Robert-Koch-Str. 27b, 48149 Münster, Germany; todescal@uni-muenster.de (L.M.T.); pethoe@uni-muenster.de (Z.P.); aschwab@uni-muenster.de (A.S.)

**Keywords:** breast cancer, Orai3, calcium, cell migration, cell adhesion, calpain, actin cytoskeleton

## Abstract

Orai3 calcium (Ca^2+^) channels are implicated in multiple breast cancer processes, such as proliferation and survival as well as resistance to chemotherapy. However, their involvement in the breast cancer cell migration processes remains vague. In the present study, we exploited MDA-MB-231 and MDA-MB-231 BrM2 basal-like estrogen receptor-negative (ER^−^) cell lines to assess the direct role of Orai3 in cell migration. We showed that Orai3 regulates MDA-MB-231 and MDA-MB-231 BrM2 cell migration in two distinct ways. First, we showed that Orai3 remodels cell adhesive capacities by modulating the intracellular Ca^2+^ concentration. Orai3 silencing (siOrai3) decreased calpain activity, cell adhesion and migration in a Ca^2+^-dependent manner. In addition, Orai3 interacts with focal adhesion kinase (FAK) and regulates the actin cytoskeleton, in a Ca^2+^-independent way. Thus, siOrai3 modulates cell morphology by altering F-actin polymerization via a loss of interaction between Orai3 and FAK. To summarize, we demonstrated that Orai3 regulates cell migration through a Ca^2+^-dependent modulation of calpain activity and, in a Ca^2+^-independent manner, the actin cytoskeleton architecture via FAK.

## 1. Introduction

Cancers are a major public health problem due to their incidence and more particularly their mortality. Among all cancers, breast cancer is one of the most diagnosed and hence one of the very frequent causes of cancer-related death in the world (2.3 million breast cancer diagnoses and 685,000 deaths in 2020 according to WHO) [1]. Ultimately, most of the patients die of metastases despite the available therapeutic progress [2,3,4].

The formation of metastasis is a complex process involving the escape of cancer cells from the primary tumor to spread and invade distant tissues. The cells must thus penetrate through their original tissue by cell migration and invasion, invade the endothelial barrier and survive in the blood and/or lymphatic circulation, escape from the circulation and finally proliferate to form a secondary tumor within a distant organ [5]. In order to prevent the formation of metastases, understanding the molecular mechanisms involved is essential.

One of the key steps during metastasis is cell migration and invasion [6,7], which are responsible for the escape of cancer cells from the primary tumor to colonize adjacent tissue. Cell migration is both a physiological and a pathophysiological cellular process. During the migration of cells, a remodeling of cytoskeleton architecture is observed as well as cycles of adhesion and detachment of cells to the substrate. At the cell front, focal adhesions anchor the cell to the substrate through mechanisms involving actin and focal adhesion remodeling [8]. On the other side, the disassembly of the focal adhesions, by calpains, permits the cell to contract and move forward [9]. In recent years, many studies have shown a dysregulation in the expression and activity of proteins involved in the turnover of focal adhesions [10,11,12].

Among all the actors involved in cell migration, calcium (Ca^2+^) plays a pivotal role [13]. Numerous studies have demonstrated the role of Ca^2+^ influx in regulating the migration of cancer cells and more specifically breast cancer cells [14,15,16]. Orai channels, whose activity is linked to the filling state of intracellular Ca^2+^ stores, are among the major players in mediating the entry of Ca^2+^ into non-excitable cells [17,18,19,20]. It has already been established that the Orai1 channel is involved in the migration of human breast cancer cells [21,22]. In addition, our team recently reported the involvement of Orai3 in cell proliferation, survival and response to chemotherapy [23,24,25]. In 2013, Motiani et al. showed that Orai3 regulates estrogen receptor-positive (ER^+^) breast cancer cell invasion and independent-anchorage cell growth [26]. However, the role of Orai3 in estrogen receptor-negative (ER^−^) breast cancer cells has not yet been tested despite the knowledge of its expression in this cell type. In the present study, we investigated the role of Orai3 in migration and adhesion of basal-type ER^−^ breast cancer cells.

We found that the Orai3 regulates the migration of two cell lines (MDA-MB-231 and MDA-MB-231 BrM2) through two different mechanisms. Orai3 regulates cell adhesion in a Ca^2+^-dependent manner by modulating the activity of calpain that in turn allows cells to detach. We demonstrated that Orai3 is active at a basal level permitting a Ca^2+^ entry leading to an increase of the intracellular Ca^2+^ concentration ([Ca^2+^]_i_) that activates calpain. Orai3 also regulates the cell morphology by rearranging the actin cytoskeleton via a Ca^2+^-independent interaction with focal adhesion kinase (FAK). Together, our results reveal new mechanisms by which Orai3 regulates breast cancer cell aggressiveness.

## 2. Material and Methods

### 2.1. Cell Culture

Human basal-like breast cancer cell lines MDA-MB-231 (MDA-231) were purchased from the American Type Culture Collection (ATCC, Molsheim, France). MDA-MB-231 BrM2 (MDA-BrM2), that specifically metastasize in the brain [27], were kindly provided by Joan Massagué (Memorial Sloan Kettering Institute, New York, NY, USA). MDA-231 shCtl and MDA-231 shOrai1 were kindly offered by Hamid Morjani (BioSpecT EA 7506, Université de Reims Champagne-Ardenne, Reims, France). Cells were grown in Eagle’s Minimal Essential Medium (EMEM; Life Technologies, Saint Aubin, France) supplemented with 20 mM HEPES, 2 mM Glutamine (Life Technologies, Saint Aubin, France) and 5% Fetal Bovine Serum (FBS, Life Technologies, Saint Aubin, France). Cells were detached using trypsin-EDTA 0.25% (Sigma, Saint-Quentin-Fallavier, France) and were incubated at 37 °C, 5% CO_2_ in a humidity-saturated atmosphere.

All experiments were performed on a collagen I matrix (2.5 μg/cm^2^). Collagen I had been extracted as previously described [28].

### 2.2. Transient Transfections

Cells were transfected with small interfering RNA (siRNA) by electroporation using the nucleofection (Amaxa Biosystems, Lonza, Aubergenville, France). MDA-231 and MDA-BrM2 cells (1.5 × 10^6^) were transiently nucleofected according to the manufacturer’s protocol with 6 µg of scrambled siRNA (siCtl) or with siRNA directed against Orai3 (siOrai3). All the experiments were performed 72 h after siRNA transfection. siRNA sequences are available in the Supplementary Material and Methods section.

Orai3 was transiently overexpressed by lipofection using the FuGENE^®^ HD Transfection Reagent (Promega, Charbonnières-les-Bains, France) according to the manufacturer’s protocol. Cells were transfected either with pEF1α-IRES-DsRed plasmid as control (referred in the text as CTL), or with pEF1α-Orai3-IRES-DsRed vector (referred to in the text as ORAI3). The Orai3 cDNA was inserted into the pEF1α-IRES-DsRed plasmid by Hasna et al. [25]. All the experiments were performed 72 h after transfection.

### 2.3. Quantitative Real-Time PCR (qRT-PCR)

Total RNA was extracted with the Trizol reagent (Sigma, Saint-Quentin-Fallavier, France) method as previously described [29]. RNA concentration and purity were determined using a spectrophotometer (NanoDrop 2000, Wilmington, NC, USA). RNA (2 μg) was converted into cDNA with the MultiScribe™ Reverse Transcriptase kit (Applied Biosystems, Carlsbad, CA, USA). Real-time PCR was performed on a LightCycler 480 System (Roche, Basel, Switzerland) using SYBR Green I PCR master mix (Life Science, Roche, Basel, Switzerland). mRNA expression was normalized to glyceraldhehyde 3-phosphate dehydrogenase (GAPDH), used as housekeeping gene, and compared to the control sample, using the Pfaffl method [30]. Primer sequences are available in Supplementary Material and Methods.

### 2.4. Western Blot Analysis

Proteins were extracted and separated as previously described [29]. The primary antibodies used were: anti-Orai3 (1:1000, Abcam, Waltham, MA, USA), anti-FAK (1: 1000, Cell Signaling Tech., Danvers, MA, USA), anti-pFAK Y397 (1:1000, Cell Signaling Tech., Danvers, MA, USA). GAPDH antibody (1:5000, Abcam, Waltham, MA, USA) was used for loading controls. Secondary antibodies were coupled to horseradish peroxidase, permitting protein detection with an enhanced chemiluminescence kit (Ozyme, Saint-Cyr-l’Ecole, France). Detection and quantification were performed as previously described [29]. All experiments were normalized to GAPDH, which was used as reference protein.

### 2.5. Cell Migration

Cell migration was quantified by means of live-cell imaging as previously described [31]. Cells were seeded 24 h prior to the experiment into 12.5 cm^2^ tissue culture flasks. These were placed into heating chambers placed on the stage of phase contrast microscopes (Axiovert 40C; Carl Zeiss, Oberkochen, Germany). Cell migration was monitored for 6 h in 5 min intervals. Image acquisition and camera (Bresser, Rhede, Germany) were controlled by MikroCamLab II software (Bresser, Rhede, Germany). Analysis by ImageJ 1.53a (National Institute of Health, Bethesda MD, USA) and Amira software (Thermofisher Scientific Corporation, Waltham, MA, USA) provided the area, perimeter and center of cells. Migration was quantified as the movement of the cell center with time. Matlab 9.2 software (Mathworks, Natick, MA, USA) was used to quantify migration speed (in μm/min) and translocation as the net distance covered during the course of experiment (in μm). Cell directionality was derived from the quotient of translocation and total track length.

Cell migration was also evaluated using a Boyden chamber model with 8 μm pore size cell culture inserts (Falcon^®^, Corning, Boulogne-Billancourt, France). Cells (4 × 10^4^) were seeded in the upper compartment in culture medium supplemented with 5% FBS. The lower compartment was filled with the same culture medium. Thus, the migration assay was performed without the addition of chemoattractant. After 24 h of incubation at 37 °C, inserts were washed with phosphate-buffered saline (PBS), then fixed with methanol for 20 min at room temperature and stained with haematoxylin for 10 min. The remaining cells on the upper side were removed from the membrane by scrubbing. Cells in 25 contiguous areas were counted at ×400 magnification. For each experiment, the number of migrating cells was normalized to their respective control (siCtl or CTL).

### 2.6. Cell Adhesion Assays

For cell adhesion assays, cells were gently detached with Versene buffer (126 mM NaCl; 5 mM KCl; 1 mM EDTA and 50 mM HEPES) 72 h post transfection, centrifuged to eliminate EDTA, and resuspended in 1 mL of culture medium. A total of 8 × 10^4^ cells were seeded in 35 mm Petri dishes and incubated at 37 °C, 5% CO_2_ (incubation time from 15 min to 8 h). Non-adherent cells were removed by smooth PBS washing after culture medium removal. Thereafter, 800 μL 3-(4,5-dimethylthiazole-2-yl)-2,5-diphenyltetrazolium bromide (MTT, Sigma, Saint-Quentin-Fallavier, France) at 0.5 mg/mL diluted in culture medium was added and incubated at 37 °C, 5% CO_2_ for 1 h. After removing MTT, 200 μL dimethylsulfoxide (DMSO, Sigma, Saint-Quentin-Fallavier, France) were added to solubilize formazan crystals. Absorbance was read at 550 nm using a microplate spectrophotometer (Infinite F200 Pro, Tecan, Lyon, France), and corresponded to adherent cell density.

### 2.7. Cell Morphology

One × 10^5^ cells were seeded in 35 mm Petri dishes 24 h prior to the experiments. Cell images were taken with an inverted optical microscope (Nikon Eclipse TS100, Leica Microsystems, Nanterre, France) with the 10× objective. Cell morphology was quantified by assessing their circularity index (CI) using ImageJ. The following formula was used to calculate CI: 4π(area)/(perimeter)^2^. CI presents a range of value from 0 to 1 where 1 corresponds to a rounded cell morphology whereas values close to 0 describe an elongated cell shape.

### 2.8. Immunofluorescence

One × 10^4^ cells were seeded on a Nunc™ Lab-Tek™ II Chamber Slide™ System (Thermofisher, Illkirch-Graffenstaden, France) 24 h prior to the immunofluorescence experiment. After washing with PBS cells were fixed with paraformaldehyde (PFA; 4%) at room temperature for 30 min. Thereafter, cells were washed and permeabilized with Triton^TM^ X-100 (0.1% in PBS; Sigma, Saint-Quentin-Fallavier, France) for 10 min. Cells were washed 3 times in PBS and blocked with 5% bovine serum albumin (Sigma, Saint-Quentin-Fallavier, France) diluted in PBS for 45 min. Phalloidin-tetramethyl rhodamine conjugate (1/1000 in PBS with 1% BSA; Santacruz Biotechnology, Dallas, TX, USA) was used to stain F-actin filaments. Cells were incubated with phalloidin-tetramethyl rhodamine conjugate in the dark for 30 min. Afterwards, cells were washed 3 times in PBS and incubated with 4′,6-diamidino-2-phenylindole (DAPI; 1%) for 1 min. Lab-Tek™ were mounted on slides using Prolong^®^ Gold antifade reagent (Life Technologies). Images were taken at 40× objective using the Zeiss Observer Z1 microscope (Carl Zeiss, Oberkochen, Germany) and analyzed in ImageJ 1.53a (National Institute of Health, Bethesda MD, USA).

### 2.9. Measurement of Calpain Activity

In order to measure calpain activity in single cells, the Boc test was used [32]. Two to three × 10^4^ cells were seeded in glass bottom dishes (Ibidi, Gräfelfing, Germany) 24 h before the experiment. Ten minutes before the experiment, the EMEM culture medium was replaced with Ringer’s solution. Subsequently, 10 μM of calpain substrate calpain 7-amino-4-chloromethylcoumarin, t-BOC-L-leucyl-L-methionine amide (CMAC, t-BOC- Leu-Met, Invitrogen) were added. After a 10 min incubation at 37 °C and in the dark, fluorescence images were taken at 40× objective using the Zeiss Observer Z1 microscope and analyzed in ImageJ. The image exposure parameters (100 ms for CMAC, t-BOC-Leu-Met) were identical in each experiment. Fluorescence intensity was measured over the entire cell area and corrected for background fluorescence in ImageJ.

### 2.10. Calcium Imaging

Ten to fifteen × 10^3^ cells were seeded on glass coverslips 24 h before each experiment. Then, cells were incubated with 3.33 μM Fura-2/AM (Sigma, Saint-Quentin-Fallavier, France) at 37 °C in the dark for 30 min. Afterwards, the cells were washed with extracellular saline solution (145 mM NaCl, 5 mM KCl, 2 mM CaCl_2_, 1 mM MgCl_2_, 5 mM glucose, 10 mM HEPES, pH 7.4). Thereafter, the coverslip was placed on the stage of a fluorescence microscope (Axiovert 200; Carl Zeiss, Oberkochen, Germany). Cells were illuminated at 340 and 380 nm using a monochromator (polychrome IV, TILL Photonics, Germany), and fluorescence emission was captured with a Cool SNAPHQ camera (Princeton Instruments, Lisses, France) after filtration through a long-pass filter (510 nm). Metafluor software (version 7.1.7.0, Molecular Devices, San Jose, CA, USA) was used for signal acquisition and data analysis. During acquisition, cells were continuously superfused with the saline solution. The store-independent Ca^2+^ entry was analyzed by following the variation of the ratio F_340_/F_380_ only by changing the Ca^2+^ concentrations of the extracellular solution from 2 mM Ca^2+^ to 0 mM Ca^2+^ (supplemented with 800 μM EGTA). The intracellular Ca^2+^ concentration is derived from the ratio of emitted fluorescence intensities for each of the excitation wavelengths (F_340_/F_380_). Store-operated Ca^2+^ entry (SOCE) was triggered by applying the classical protocol using 1 μM thapsigargin for Ca^2+^ store depletion which lead to Ca^2+^ influx through store-operated channels (SOC).

To estimate divalent cation influx under basal conditions, we used the manganese (Mn^2+^) quenching technique as previously described [23]. After Fura2/AM loading and washings, cells were excited at 360 nm and fluorescence was recorded at 510 nm. After 2 min, the Ca^2+^ (2 mM) present in the superfusion solution was replaced by 2 mM Mn^2+^ solution. The Mn^2+^ influx, a corroborate of Ca^2+^ influx, was estimated from the quenching rate of fluorescence at 360 nm. The Mn^2+^ quenching extracellular solution was composed of 145 mM NaCl, 5 mM KCl, 2 mM MnCl_2_, 1 mM MgCl_2_, 5 mM glucose and 10 mM HEPES (pH 7.4).

### 2.11. Proximity Ligation Assay

One × 10^4^ cells were seeded on Nunc™ Lab-Tek™ II Chamber Slide™ System (Thermofisher, Illkirch-Graffenstaden, France) 24 h prior to the proximity ligation assay (PLA) experiment. After washings with PBS, cells were fixed with PFA (4%) at room temperature for 30 min. Thereafter, cells were washed and permeabilized with Triton^TM^ X-100 (0.1% in PBS; Sigma, Saint-Quentin-Fallavier, France) for 10 min. The Duolink in situ PLA detection kit (Sigma-Aldrich, Saint-Quentin-Fallavier, France) was used to detect interactions between FAK and Orai3. Experiments were performed following the manufacturer’s instructions. Primary antibodies were incubated at 1:500 overnight at 4 °C. Red fluorescent oligonucleotides produced as the end product of the procedure were visualized using the Zeiss Observer Z1 microscope (Carl Zeiss, Oberkochen, Germany). Images were analyzed using ImageJ software 1.53a (National Institute of Health, Bethesda, MD, USA).

### 2.12. Statistical Analysis

All data are expressed as mean ± SEM (standard error of the mean) of at least three independent experiments. N refers to number of experiments and n refers to the number of cells. The mean values of two groups were compared by the student’s *t*-test and the mean values of more than two groups were compared using two-way analysis of variance (ANOVA), using GraphPad Prism 7.0 software (La Jolla, CA, USA). The statistical significance *p* < 0.05, *p* < 0.01 and *p* < 0.001 are represented as *, ** and ***, respectively.

## 3. Results

### 3.1. The Orai3 Ca^2+^ Channel Regulates Cell Migration and Is Activated at a Basal Level inMDA-231 and MDA-BrM2 Breast Cancer Cells

**Figure 1:**
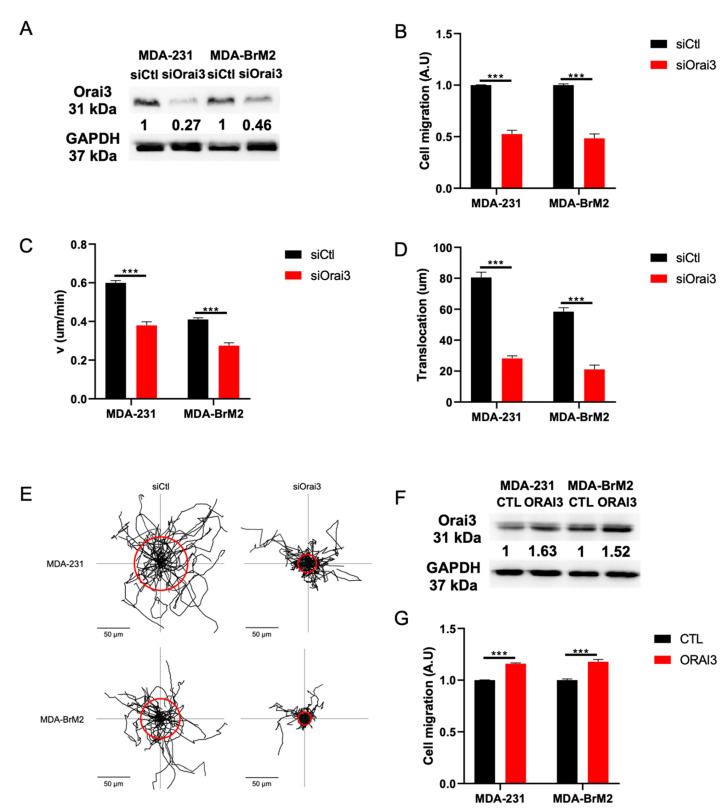
Orai3 regulates basal-like breast cancer cell migration. (**A**) Orai3 expression evaluated by Western Blot 72 h post transfection (N = 3; *** *p* < 0.001). (**B**) Orai3 activity regulates MDA-231 and MDA-BrM2 cell migration. Cell migration experiments were performed using Boyden chambers (N = 3; *** *p* < 0.001). (**C**,**D**) Both cell migration speed (**C**) and translocation (**D**) are impaired by siOrai3. Summary of the migration experiments shown in E and evaluated by live-cell imaging (MDA-231 siCtl n = 49; MDA-231 siOrai3 n = 43; MDA-BrM2 siCtl n = 39; MDA-BrM2 siOrai3 n = 42; N = 3; *** *p* < 0.001). (**E**) Trajectories of migrating cells in siCtl and siOrai3 MDA-231 and MDA-BrM2 cells. The radius of the red circles illustrates the mean translocation of each condition (Scale bar: 50 μm). (**F**,**G**) Orai3 overexpression increases protein expression levels (**F**) as well as MDA-231 and MDA-BrM2 cell migration (**G**). Cell migration experiments were performed using Boyden chambers (N = 3; *** *p* < 0.001).

Until now, no study has been conducted on the role of the Orai3 Ca^2+^ channel in the migration of cancer cells. In order to understand the mechanisms by which Orai3 regulates migration we studied the impact of the downregulation of Orai3 by siRNA on cell migration in two aggressive breast cancer cell lines namely MDA-MB-231 (MDA-231) and MDA-MB-231 BrM2 (MDA-BrM2). Silencing of Orai3 decreases Orai3 protein levels by 77.0 ± 9.1 and 54.0 ± 3.1% in MDA-231 and MDA-BrM2 cells, respectively (Figure 1A and Figure S1A). siOrai3 decreases mRNA levels by 87% in MDA-231 cells and 82% in MDA-231 BrM2 cells (Figure S1B). Furthermore, Orai3 silencing did not affect the expression of STIM1, STIM2 and Orai1 at both mRNA and protein levels (Figure S1C–G).

Using a Boyden chamber migration test, we showed a reduction in cell migration by 50.0 ± 5.6% and 55.0 ± 6.1% in MDA-231 and MDA-BrM2 cells transfected with siOrai3, respectively (Figure 1B). These data were confirmed by a MTT test which allowed us to rule out any effect due to proliferation because the silencing of Orai3 failed to affect both MDA-231 and MDA-BrM2 cell viability and proliferation (Figure S2A–C). In order to confirm the results obtained by the Boyden chamber model, we assessed cell migration of the two cell lines also by means of live-cell imaging. Thereby, we determined migration parameters such as cell migration speed, translocation and directionality. We show that the migration speed of siOrai3 MDA-231 and siOrai3 MDA-BrM2 cells are reduced by 34.0 ± 3.9% and 38.0 ± 3.7%, respectively, when compared to the siCtl condition (Figure 1C). Moreover, we observed a decrease in translocation by 65.0 ± 3.2% and 63.0 ± 2.7% in both cell lines (MDA-231 and MDA-BrM2, respectively) (Figure 1D,E). We also observed a decrease in cell migration directionality, by 48.0 ± 3.4% and 48.0 ± 3.1% of siOrai3 MDA-231 and siOrai3 MDA-BrM2 cells, respectively (Figure S2D). We have also studied the effect of transient overexpression of Orai3 on cell migration. By Western Blot, Orai3 overexpression showed an increase of protein levels by 63 ± 2.7% in MDA-231 cells and 52 ± 1.1% in MDA-BrM2 cells (Figure 1F and Figure S2E). Orai3 overexpression was also validated at the mRNA level in both cell lines (Figure S2F). Using a Boyden chamber model we found an increase of cell migration by 15 ± 0.9% and 17 ± 2.1% in both cell lines compared to CTL (Figure 1G). The cell viability was not affected (Figure S2G).

**Figure 2:**
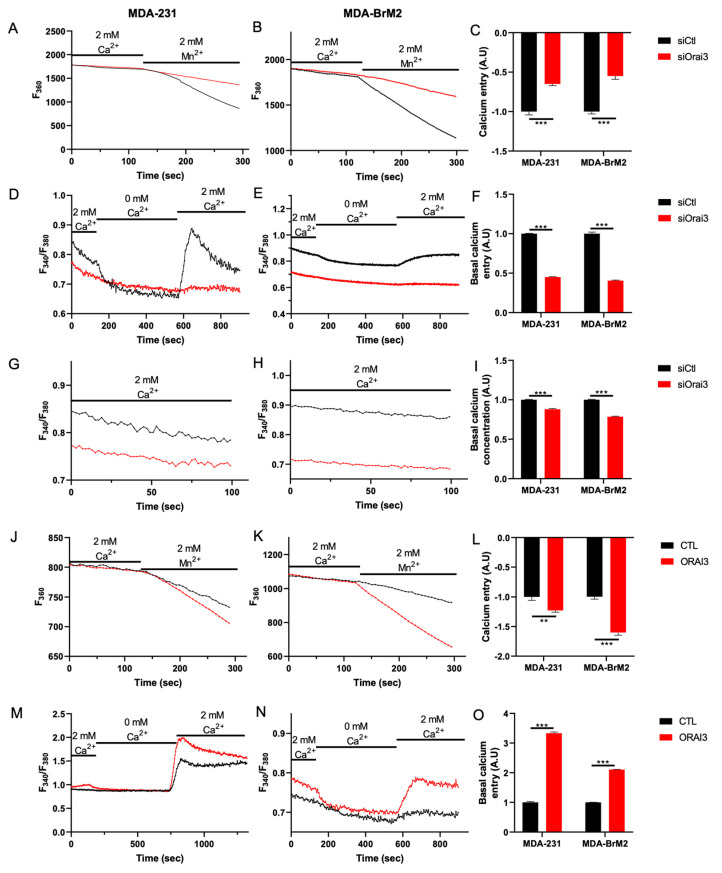
Orai3 is active at a basal level and modulates the intracellular Ca^2+^ concentration. (**A–C)**. Traces (**A**,**B**) and quantification (**C**) of Mn^2+^ quenching measured at F_360_ in MDA-231 (**A**,**C**) and MDA-BrM2 (**B**,**C**) cell lines transfected with siCtl or siOrai3 (MDA-231 siCtl n = 152; MDA-231 siOrai3 n = 162; MDA-BrM2 siCtl n = 212; MDA-BrM2 siOrai3 n = 228; N = 3; *** *p* < 0.001). (**D**–**F**). Traces (**D**,**E**) and quantification (**F**) of basal Ca^2+^ entry measured by the calculation of the F_340_/F_380_ in MDA-231 (**D**,**F**) and MDA-BrM2 (**E**,**F**) cell lines transfected with siCtl or siOrai3 (MDA-231 siCtl n = 269; MDA-231 siOrai3 n = 255; MDA-BrM2 siCtl n = 222; MDA-BrM2 siOrai3 n = 322; N = 3; *** *p* < 0.001). (**G**–**I**). Ca^2+^ concentration plot (**G**,**H**) and quantification (**I**) measured by the calculation of the F_340_/F_380_ in MDA-231 (**G**,**I**) and MDA-BrM2 (**H**,**I**) cell lines transfected with siCtl or siOrai3 (MDA-231 siCtl n = 423; MDA-231 siOrai3 n = 441; MDA-BrM2 siCtl n = 404; MDA-BrM2 siOrai3 n = 490; N = 3; *** *p* < 0.001). (**J**–**L**). Traces (**J**,**K**) and quantification (**L**) of Mn^2+^ quenching measured at F_360_ in MDA-231 (**J**,**L**) and MDA-BrM2 (**K**,**L**) cell lines transfected with CTL or ORAI3 (MDA-231 CTL n = 182; MDA-231 ORAI3 n = 169; MDA-BrM2 CTL n = 198; MDA-BrM2 ORAI3 n = 158; N = 3; ** *p* < 0.01; *** *p* < 0.001). (**M**–**O**). Traces (**M**,**N**) and quantification (**O**) of basal Ca^2+^ entry measured by the calculation of the F_340_/F_380_ in MDA-231 (**M**,**O**) and MDA-BrM2 (**N**,**O**) cell lines transfected with CTL or ORAI3 (MDA-231 CTL n = 212; MDA-231 ORAI3 n = 209; MDA-BrM2 CTL n = 188; MDA-BrM2 ORAI3 n = 199; N = 3; *** *p* < 0.001).

We next studied the impact of siRNA against Orai3 on Ca^2+^ entry using the manganese-quenching technique. Orai3 silencing decreases the Mn^2+^ quench by 40.0 ± 2.1% and 45.0 ± 3.6% in MDA-231 and MDA-BrM2 cells, respectively (Figure 2A–C). Moreover, we used the basal Ca^2+^ entry protocol to show that entry of cations is supported by entry of Ca^2+^. Orai3 silencing decreases basal Ca^2+^ entry (assessed as the F_340_/F_380_ ratio) by 57.0 ± 1.1 and 62.0 ± 1.3% in MDA-231 and MDA-BrM2 respectively (Figure 2D–F). This basal Ca^2+^ entry also regulates the intracellular Ca^2+^ concentration. Indeed, we observed a 13.0 ± 0.6 and 18.0 ± 0.4% decrease in the intracellular Ca^2+^ concentration when the expression of the Orai3 channel was reduced (Figure 2G–I). Furthermore, using the classical SOCE protocol, we confirmed that Orai3 does not participate in SOCE (Figure S3A–C) in our breast cancer cell lines. Besides, we showed that Orai3 overexpression increases the cation entry by 23.0 ± 3.6 and 62.0 ± 1.6% in MDA-231 and MDA-BrM2 cells, respectively (Figure 2J–L). We also found that basal Ca^2+^ entry increased by 3.25-fold and 2.15-fold when Orai3 was overexpressed in MDA-231 and MDA-BrM2, respectively (Figure 2M–O).

### 3.2. Orai3 Regulates Cell Migration in a Ca^2+^-Dependent Manner via a Modulation of Cell Adhesive Capacities

**Figure 3:**
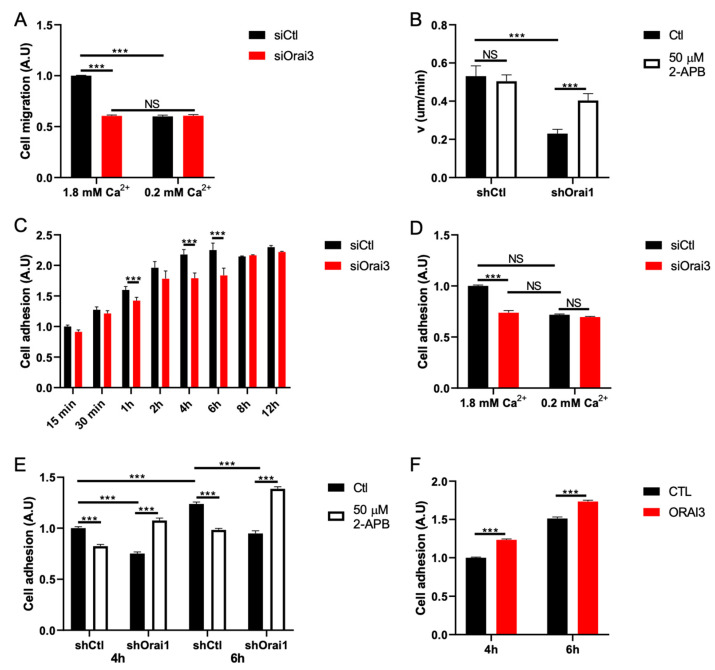
Orai3 regulates cell migration and adhesion in a Ca^2+^-dependent manner. (**A**,**B**). Orai3-mediated Ca^2+^ entry supports cell migration. Migration assay using Boyden chambers in the presence or absence of Ca^2+^ (**A**) (N = 3; *** *p* < 0.001; NS Non-Significant) and cell migration speed following Orai3 activation with 2-APB, evaluated by live-cell imaging (**B**) (shCtl Ctl n = 39; shCtl 2-APB n = 41; shOrai1 Ctl n = 42; shOrai1 2-APB n = 39; N = 3; *** *p* < 0.001; NS Non-Significant). (**C**). Orai3 activity regulates cell adhesion. Cell adhesion assay using an MTT-based technique (N = 3; *** *p* < 0.001). (**D**,**E**). Cell adhesion is mediated by Ca^2+^ entry through Orai3. MTT-based cell adhesion assay in the presence or absence of Ca^2+^ (**D**) and following Orai3 activation with 2-APB (**E**) (N = 3; *** *p* < 0.001; NS Non-Significant). (**F**). MTT-based cell adhesion assay showing that Orai3 overexpression enhances cell adhesion (N = 3; *** *p* < 0.001; NS Non-Significant).

Given the ability of Orai3 to regulate basal Ca^2+^ entry and in order to understand whether Ca^2+^ is involved in cell migration, we cultured cells in normal medium (1.8 mM Ca^2+^—normal Ca^2+^) and in a medium poor in Ca^2+^ (0.2 mM Ca^2+^—low Ca^2+^). Orai3 silencing reduced cell migration of MDA-231 cells by 45.0 ± 0.9% under normal Ca^2+^ conditions (Figure 3A). Reducing the concentration of extracellular Ca^2+^ (low Ca^2+^) decreased cell migration to a similar extent, i.e., by 41.0 ± 1.3%. This effect of low extracellular Ca^2+^ is not enhanced by siOrai3 (Figure 3A). Similar results were obtained on the MDA-BrM2 cell line (Figure S4A). Orai3 knockdown under both normal Ca^2+^ and low Ca^2+^ conditions reduced MDA-BrM2 cell migration by 44.0 ± 1.8% (Figure S4A). As expected, siOrai3 in low Ca^2+^ does not show any additional decrease in cell migration (Figure S4A). In all cases, siOrai3 does not affect cell viability (Figure S4B,C). All together, these results argue for a regulation of MDA-231 and MDA-BrM2 cell migration by Orai3-mediated basal Ca^2+^ entry.

Very few pharmacological agents are known to modulate the Orai3 channel. Among them, 2-APB had been shown to activate Orai3 when used at 50 μM [33,34]. However, at this concentration, 2-APB inhibits Orai1 activity [34]. Besides, past studies reported the involvement of Orai1 in MDA-231 cell migration [22]. Given these data, we chose to use MDA-231 cell line stably transfected with shOrai1 to avoid any bias due to 2-APB-mediated Orai1 inhibition (Figure S4D). Our results showed that shOrai1 cells exhibit a cell migration speed reduced by 68.0 ± 3.6% compared to shCtl cells (Figure 3B). Incubation of the cell with 2-APB (50 μM) during 6 h has no effect on shCtl cells (Figure 3B). Interestingly, 2-APB treatment increases cell migration speed by 90.0 ± 10% in shOrai1 cells compared to untreated shOrai1 cells (Figure 3B).

During cell migration, adhesion plays a pivotal role [35,36]. First, we studied cell adhesion using a kinetic protocol from 15 min to 12 h of cell adhesion in MDA-231 (Figure 3C) and MDA-BrM2 (Figure S4D) cell lines. Orai3 silencing decreased MDA-231 cell adhesion by 21.0 ± 4.1 and 18.0 ± 4.8% at 4 h and 6 h, respectively (Figure 3C). The corresponding values for the MDA-BrM2 cell line are a decrease of 18.0 ± 2.1%, 15.0 ± 4.1%, 23.0 ± 3.3% and 29.0 ± 4.7% after 1 h, 2 h, 4 h and 6 h, respectively (Figure S4D). After 6 h of cell adhesion, we did not observe any differences in cell adhesion neither in MDA-231 (Figure 3C) nor in MDA-BrM2 (Figure S4D) cells transfected with siOrai3 when compared to their respective siCtl cells. We therefore chose 4 h and 6 h cell adhesion times in order to investigate the impact of a reduced extracellular Ca^2+^ concentration. We showed that the reduction in cell adhesion in low Ca^2+^ medium (by 28.0 ± 0.7%) is similar to that measured in cells transfected with siOrai3 and grown in 1.8 mM Ca^2+^ (25.0 ± 1.9%) (Figure 3D). Moreover, siOrai3 does not affect cell adhesion when cells are grown in the low Ca^2+^ medium (Figure 3D). Similar results were obtained in the MDA-BrM2 cell line (Figure S4F).

To support these results, we incubated shOrai1 MDA-231 cells in the presence of 2-APB (50 μM) for 4 h and 6 h and measured the adhesion rate. We found that 2-APB increased adhesion of MDA-231 shOrai1 cells by 29.0 ± 1.7% at 4 h and by 33.0 ± 0.9% at 6 h (Figure 3E). Furthermore, overexpression of Orai3 in the MDA-231 cell line increases cell adhesion by 23.0 ± 1.1% and 17.0 ± 2.3% at 4 h and 6 h, respectively (Figure 3F). Orai3 overexpression in the MDA-BrM2 cell line also increased cell adhesion by 25.0 ± 6.1% and 18.0 ± 3.2% at 4 h and 6 h, respectively (Figure S4G).

### 3.3. Orai3, by Regulating Ca^2+^ Entry, Controls Calpain Activity

**Figure 4:**
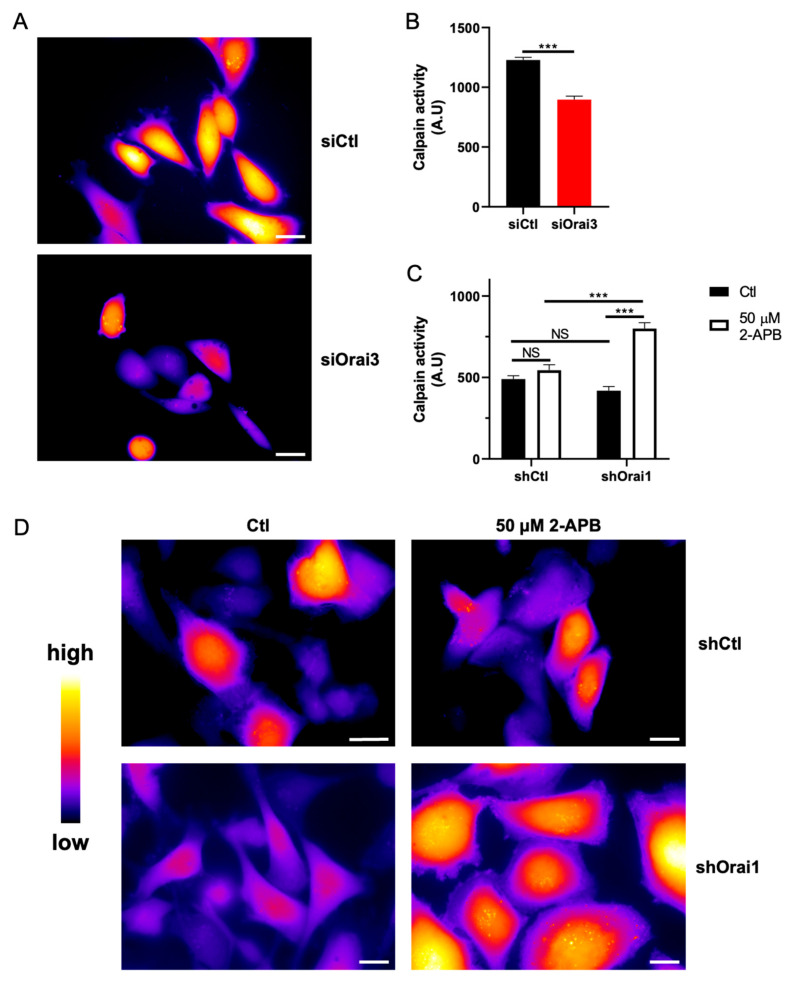
Orai3 silencing impairs MDA-231 cell adhesion. (**A**,**B**). Orai3 silencing decreased calpain activity. Representative fluorescence images (**A**) and fluorescence quantification (**B**) of calpain substrate CMAC, t-BOC-Leu-Met (siCtl n = 209; siOrai3 n = 211; N = 3; *** *p* < 0.001). (**C**,**D**). Orai3 activation with 2-APB enhanced calpain activity. Measurement (**C**) and representative fluorescence pictures (**D**) of calpain substrate CMAC, t-BOC-Leu-Met (shCtl Ctl n = 243; shCtl 2-APB n = 201; shOrai1 Ctl n = 233; shOrai1 2-APB n = 211; N = 3; *** *p* < 0.001; NS Non-Significant). Scale bar: 10 μm.

Among Ca^2+^-dependent actors involved in cell migration, calpains are known to play a major role in cell migration [37]. Since calpain contributes to the remodeling of focal adhesions, this process becomes Ca^2+^-dependent [38]. We therefore sought to understand whether the decrease in intracellular Ca^2+^ due to siOrai3 is associated with a decrease in the activity of calpain. Using the fluorescent calpain substrate CMAC, t-BOC-Leu-Met, we found that siOrai3 reduced calpain activity by 25.0 ± 4.4% in the MDA-231 cell line (Figure 4A,B) and by 38.0 ± 3.2% in the MDA-BrM2 cell line (Figure S5A,B) when compared to siCtl. To go further, we have also shown that treatment with 2-APB (50 μM) increased the activity of calpain in MDA-231 shOrai1 cells by almost 50.0 ± 6.9% (Figure 4C,D). Furthermore, we employed an atomic force microscope to evaluate adhesion forces in MDA-231 and MDA-BrM2 cell lines within the first 10 s of cell-matrix contact by means of single-cell force spectroscopy. We have found that siOrai3 has no impact on the initial adhesion forces in both cell lines (Figure S5C,D).

### 3.4. Orai3 Maintains an Elongated Cell Morphology through a Ca^2+^-Independent Mechanism

**Figure 5:**
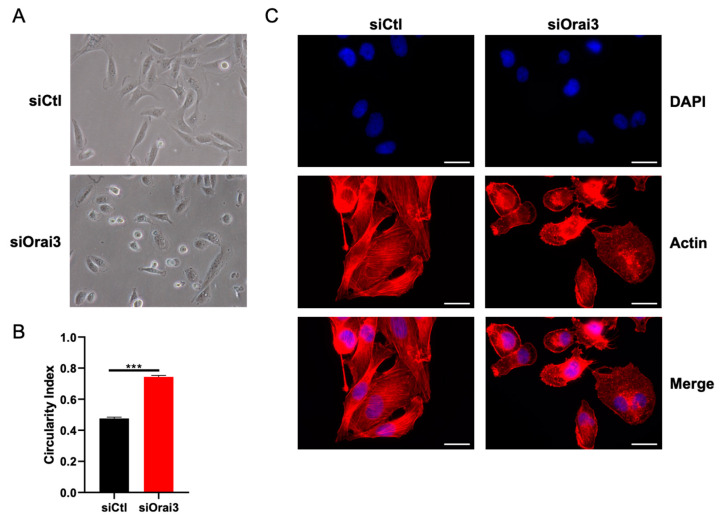
Orai3 modulates cell morphology. (**A**,**B**). siOrai3 MDA-231 cells present a rounded morphology. Representative images (**A**) and calculation of the circularity index (**B**) of MDA-231 cell morphology (siCtl n = 246; siOrai3 n = 312; N = 3; *** *p* < 0.001). (**C**). Actin cytoskeleton architecture depends on Orai3 expression levels. Fluorescent rhodamine phalloidin staining reveals the actin architecture of siCtl and siOrai3 MDA-231 cells (N = 3). Scale bar: 10 μm.

Cell migration is also governed by a rearrangement of the cytoskeleton and more particularly by cycles of polymerization and depolymerization of actin filaments which in turn affect the cell morphology [39,40]. Thus, we first studied the cell morphology of MDA-231 and MDA-BrM2 cells transfected with siCtl or siOrai3. MDA-231 cells transfected with siOrai3 exhibited a more rounded phenotype compared to siCtl cells. The circularity index (CI) of siOrai3 MDA-231 cells amounts to 0.75 ± 1.3, while cells transfected with siCtl have a circularity index of 0.49 ± 0.8 (Figure 5A,B). Similar results were obtained for the MDA-BrM2 cell line. The circularity index of siCtl BrM2 cells is 0.57 ± 1.5 and 0.79 ± 0.4 in siOrai3 MDA-BrM2 cells (Figure S6A,B).

Many studies reported an involvement of F-actin in regulating cell morphology [40,41]. Therefore, we hypothesized a possible participation of F-actin polymerization in determining the morphology of both cell lines transfected or not with siOrai3. Using fluorescence microscopy, we showed that the Orai3 downregulation causes an alteration in the polymerization of F-actin. MDA-231 (Figure 5C) as well as MDA-BrM2 (Figure S6C) cells transfected with a siCtl exhibit fibers formed from F-actin while the cells lacking Orai3 do not have this F-actin architecture.

Furthermore, Orai3 overexpression does not further modify cell morphology of both cell lines (Figure S6D,E).

**Figure 6:**
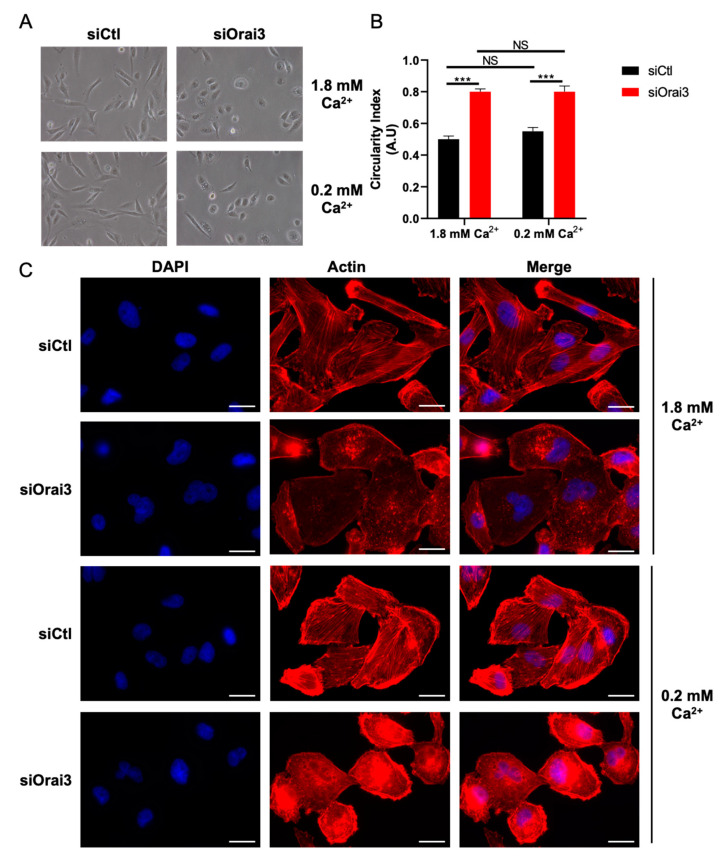
Cell morphology is maintained by the presence of Orai3 and not its activity. (**A**,**B**). The presence, not the activity of Orai3 regulates cell morphology. Representative images (**A**) and calculation of the circularity index (**B**) of MDA-231 morphology (siCtl 1.8 mM n = 207; siOrai3 1.8 mM n = 211; siCtl 0.2 mM n = 209; siOrai3 0.2 mM n = 198; N = 3; *** *p* < 0.001; NS Non-Significant). (**C**) Fluorescent rhodamine phalloidin staining showing the actin architecture of siCtl and siOrai3 MDA-231 cells cultivated in 1.8 mM or 0.2 Ca^2+^ medium (N = 3). Scale bar: 10 μm.

Since cell migration of MDA-231 and MDA-BrM2 cells is Ca^2+^-dependent, we evaluated the potential role of Ca^2+^ in the morphological changes regulated by Orai3. We thus cultured the cells in a normal (1.8 mM) or in low Ca^2+^ medium (0.2 mM). To our surprise, siCtl MDA-231 cells transfected with siCtl and cultured in a low Ca^2+^ medium do not change their morphology (CI siCtl 1.8 mM = 0.49 ± 3.2; CI siCtl 0.2 mM = 0.52 ± 3.5). Moreover, siOrai3 MDA-231 cells cultured in low Ca^2+^ medium exhibit a similar cell morphology when compared to the siOrai3 cells cultured in normal Ca^2+^ medium (CI siOrai3 1.8 mM = 0.81 ± 1.8; CI siOrai3 0.2 mM = 0.83 ± 4.2) (Figure 6A,B). The morphology changes are supported by the polymerization of F-actin. Indeed, siCtl cells cultured in both normal and low Ca^2+^ conditions showed F-actin filaments. On the contrary, siOrai3 cells cultured in both culture media display a modified actin architecture compared to siCtl cells (Figure 6C). In siCtl MDA-BrM2 cells, we observed a small but significant increase of the circularity index under low Ca^2+^ medium conditions (CI of 0.63 ± 2.9 while the cells cultured in normal Ca^2+^ medium have a CI of 0.55 ± 1.7). Silencing of Orai3 induced similar morphological changes in both culture media (IC siOrai3 1.8 mM = 0.77 ± 2.3; IC siOrai3 0.2 mM = 0.79 ± 0.9) (Figure S7A,B). Regarding F-actin architecture, similar to siOrai3 MDA-231 cells, siOrai3 MDA-BrM2 cells also exhibit an altered actin architecture (in normal and low Ca^2+^) while siCtl MDA-BrM2 cells show long F-actin filaments (Figure S7C).

### 3.5. Orai3 Interacts with FAK and Regulates Its Expression

**Figure 7:**
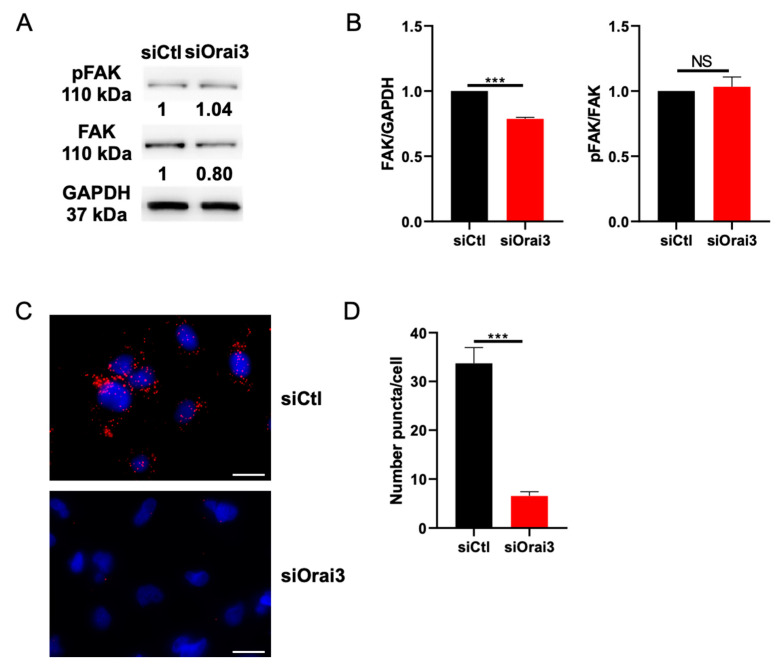
Orai3 modulates and interacts with focal adhesion kinase (FAK). (**A**,**B**). Involvement of Orai3 in FAK total expression. Representative Western blot (**A**) and quantification (**B**) of FAK expression and FAK phosphorylation in MDA-231 cells (N = 3; *** *p* < 0.001; NS Non-Significant). (**C**,**D**). Orai3 interacts with FAK. Representative immunofluorescences pictures (**C**) and Quantification (**D**) of the interaction between FAK and Orai3 (siCtl n = 341; siOrai3 n = 312; N = 3; *** *p* < 0.001). Scale bar: 10 μm.

One of the most well-known proteins that regulates cell adhesion processes as well as the polymerization of actin is the FAK protein [42,43]. We therefore evaluated the expression of this protein and its phosphorylation in MDA-231 and MDA-BrM2 cells transfected with siCtl or siOrai3. Using Western Blot analysis, we showed that the cells lacking Orai3 channel have a reduced FAK expression: 32.0 ± 1.3% less in MDA-231 cells (Figure 7A,B) and 35.0 ± 2.9% less in MDA-BrM2 cells (Figure S8A) when compared to their respective controls. Interestingly, even though total FAK expression decreased, the phosphorylation rate of FAK shows no differences in siOrai3 compared to siCtl (Figure 7B). Similar results were obtained on Orai3-overexpressing MDA-231 cells (Figure S8E).

We therefore studied a possible interaction of Orai3 with FAK using the co-immunoprecipitation and the PLA techniques. Indeed, interactions between ion channels and FAK leading to its activation have been reported [44]. We first showed that FAK and Orai3 form an interacting complex in MDA-231 and MDA-BrM2 cells (Figure S8B). To verify this interaction, we used the PLA technique. During this experiment, we assessed whether FAK and Orai3 could directly interact with each other. Our results showed that Orai3 and FAK are located very close to each other (<40 nm) and can possibly interact. We counted an average of 33 ± 4 amplification puncta per cell in siCtl cells while we counted only 7 ± 1 in siOrai3 MDA-231 cells (Figure 7C,D). Likewise, a similar result was obtained in the MDA-BrM2 cell line (siCtl: 48 ± 2 puncta per cell; siOrai3: 10 ± 1 puncta) (Figure S8C,D). FAK is pivotal in the formation of focal adhesions which in turn are necessary for cell migration and signaling leading to F-actin polymerization. We therefore also evaluated the expression of another major component of focal adhesions, the β1 integrin. Our results showed that β1 integrin expression following transfection with siOrai3 decreased by 32 ± 2.1% in MDA-231 cells and by 29 ± 4.3% in MDA-BrM2 cells (Figure S8F,G).

## 4. Discussion

Ca^2+^ channels, in particular Orai channels, have important roles in regulating biological processes [45,46]. The dysregulation of their expression and/or activity is involved in the control of different traits of breast cancer behavior such as proliferation, apoptosis and resistance to chemotherapy [23,24,25,47]. Here, we focused on studying the role of the Orai3 channel in the cell migration mechanisms of the most aggressive type of breast cancer.

The key findings of our study are: (i) Orai3 regulates basal-like breast cancer cell migration via a modulation of adhesive capacities. This likely occurs through a regulation of the basal Ca^2+^ entry and intracellular Ca^2+^ concentration. (ii) Orai3 regulates cell morphology and polymerization of F-actin filaments by interacting with FAK.

Orai3 has mainly been studied in ER^+^ breast cancer cell lines where it participates in the SOCE [23]. However, very few studies have been conducted to understand the role of Orai3 in the basal-like ER^−^ type breast cancer cell lines. Motiani et al. showed in 2010 that Orai1 (and not Orai3) is one of the major components in the regulation of SOCE in the MDA-231 line [48]. Recently, Monteith’s team made it clear that Orai3 does not constitute a SOC in the ER^−^ breast cancer cell line MDA-MB-468. In this work, we confirm that Orai3 is not involved in SOCE [49] in ER^−^ breast cancer cell lines MDA-231 and MDA-BrM2. We show for the first time that this channel participates in the basal entry of Ca^2+^ into these two ER^−^ cell models. Besides, we show that Orai3-mediated Ca^2+^ entry participates in the regulation of the intracellular Ca^2+^ concentration.

Here, we demonstrated the involvement of Orai3 in the regulation of the migration of MDA-231 and MDA-BrM2 cell lines. Orai3 silencing decreases cell migration by approximately 50% in these cells. This effect is not linked to an impaired viability of the cells. Our results suggest a specific effect of Orai3 depending on the ER status of breast cancer cells. In ER^+^ luminal breast cancer cells this channel regulates proliferation and survival [23,24], whereas in ER^−^ basal-like breast cancer cells Orai3 rather regulates migration without affecting cell proliferation and/or survival.

The involvement of Orai3 in the migration of MDA-231 and MDA-BrM2 cells requires both the channel protein and the channel function. The fact that migration and adhesion of siOrai3 cells cultured in normal Ca^2+^ medium is not different from that of cells cultured in low Ca^2+^ medium suggests the involvement of a common pathway of Orai3 and Ca^2+^ in the regulation of these two cellular processes. Cell migration is a dynamic process requiring an interaction between the cell and the extracellular matrix through focal adhesions [50]. The remodeling of the latter is essential for cell migration. In this context the process of deadhesion is as essential as cell adhesion [51]. In the late 1990s a family of proteins, calpains, were reported to have a significant role in cell migration and adhesion [52,53,54]. Here, we found that Orai3, by its channel function, drives cell migration and adhesion processes by modulating calpain activity. Interestingly, similarly to our results, it has been shown in MDA-MB-435S (an ER^−^ breast cancer cell line) that stores independent Ca^2+^ entry through Orai1, in association with potassium channel SK3 (K_Ca_2.3), tunes calpain activation to steer cell migration [55,56]. Here we bring a new aspect of how Orai channels regulate cell migration. Orai1 supports cell migration of MDA-231 cells by mediating SOCE [22], while Orai3 drives cell migration via its basal activity and a modulation of calpain activity. However, further studies are needed for understanding molecular pathways involved in Orai3-mediated calpain activation.

During cell migration actin holds a special place. Actin, in the form of stress fibers, provides most of the mechanical forces involved in cell migration [57]. Cell migration necessitates actin remodeling [58]. Here we show a modification of the F-actin cytoskeleton when Orai3 is downregulated. In fact, the F-actin polymerization is altered in the absence of Orai3. This disorganization is the basis for the rounded morphology of the MDA-231 and MDA-BrM2 cells transfected with siOrai3. To our surprise, the morphological changes of siOrai3 cells, in particular of MDA-231 cells, is independent of the ambient Ca^2+^ concentration. Regarding the MDA-BrM2 cell line, we also reported a slight regulation of the architecture of the actin cytoskeleton by Ca^2+^ as was reported by Pardo-Pastor et al. [41]. They showed that the PIEZO2-mediated Ca^2+^ entry facilitates the architecture of the actin cytoskeleton.

F-actin polymerization is regulated by many factors including FAK [59]. FAK is a tyrosine kinase controlling various cellular processes including cell migration as well as the formation and remodeling of focal adhesions [60] and promoting actin dynamics [42,43]. We found that siOrai3 decreases total FAK expression by 32% in MDA-231 and 35% in MDA-BrM2 cells without any change in the amount of phosphorylated FAK.

Consistent with our results, several studies have reported the pivotal role of FAK in actin polymerization [61,62,63]. Also, FAK requires phosphorylation to mediate actin remodeling [64]. Since we found no Orai3-dependent changes in FAK phosphorylation, we suggest that Orai3 may interact with FAK and regulate its expression in order to support FAK turnover, and thus initiate signaling that leads to F-actin polymerization. Such an interaction between FAK and ion channels, particularly potassium channels, has already been reported [44,65]. Clearly, further studies are needed regarding the mechanisms of interaction between Orai3 and FAK.

In conclusion, we show that Orai3 mediates basal Ca^2+^ entry in basal-like breast cancer cells. Orai3 regulates breast cancer cell migration through two mechanisms: (i) through a regulation of calpain activity and cell adhesion by regulating intracellular Ca^2+^ concentration, and (ii) via the remodeling of actin cytoskeleton architecture by regulating FAK expression, probably through a Ca^2+^-independent mechanism.

## Data Availability

The study did not report any data.

## Supplementary Materials

The following are available online at https://www.mdpi.com/article/10.3390/cells10123487/s1. Table S1: siRNA sequences. Table S2: Primers sequences. Table S3: Primary antibodies, dilution and manufacturer. Figure S1: A. Quantification of Orai3 expression evaluated by Western blot represented in Figure 1A (N = 3; *** *p* < 0.001). B. Orai3 expression evaluated by qPCR 72 h after transfection with siOrai3 in MDA-231 and MDA-BrM2 cell lines (N = 3; *** *p* < 0.001). C–E. Orai1, STIM1 and STIM2 expression in MDA-231 (C,D) and MDA-BrM2 (C,E) cell lines evaluated by Western blot 72 h after transfection with siOrai3 (N = 3; NS Non Significant). F,G. Orai1, Orai2, STIM1 and STIM2 expression in MDA-231 (F) and MDA-BrM2 (G) cell lines evaluated by qPCR 72h after transfection with siOrai3 (N = 3; NS Non Significant). Figure S2: A,B. Cell proliferation assay, using MTT-based assay, in MDA-231 (A) and MDA-BrM2 (B) cells transfected with siOrai3 (N = 3; NS Non Significant). C. Cell viability assay, using MTT-based assay, in MDA-231 and MDA-BrM2 cells transfected with siOrai3 (I) (N = 3; NS Non-Significant). D. Cell directionality of MDA-231 and MDA-BrM2 cell lines transfected with siCtl or siOrai3 (MDA-231 siCtl n = 49; MDA-231 siOrai3 n = 43; MDA-BrM2 siCtl n = 39; MDA-BrM2 siOrai3 n = 42; N = 3; *** *p* < 0.001). E. Quantification of Orai3 overexpression evaluated by Western blot and represented in Figure 1F (N = 3; *** *p* < 0.001). F. Orai3 overexpression in MDA-231 and MDA-BrM2 cell lines evaluated by qPCR 72h after transfection with ORAI3 (N = 3; *** *p* < 0.001). G. Cell viability assay, using MTT-based assay, in MDA-231 and MDA-BrM2 cells transfected with ORAI3 (N = 3; NS Non-Significant). Figure S3: A–C. Traces (A,B) and quantification (C) of SOCE measured by the calculation of the F_340_/F_380_ in MDA-231 (A,C) and MDA-BrM2 (B,C) cell lines transfected with siCtl or siOrai3 (MDA-231 siCtl n = 198; MDA-231 siOrai3 n = 156; MDA-BrM2 siCtl n = 134; MDA-BrM2 siOrai3 n = 142; N = 3; *** *p* < 0.001). D/E/F. Traces (D,E) and quantification (F) of basal Ca^2+^ entry measured by the calculation of the F_340_/F_380_ in MDA-231 (D,F) and MDA-BrM2 (E,F) cell lines transfected with siCtl or siOrai1 (MDA-231 siCtl n = 90; MDA-231 siOrai3 n = 88; MDA-BrM2 siCtl n = 94; MDA-BrM2 siOrai3 n = 89; N = 3; *** *p* < 0.001). Figure S4: A. Migration assay using Boyden chambers with MDA-BrM2 cells transfected with siOrai3 in the presence or absence of Ca^2+^ (A) (N = 3; *** *p* < 0.001; NS Non-Significant). B,C. Cell viability assay, using MTT-based assay, in MDA-231 (B) and MDA-BrM2 (C) cells transfected with siOrai3 (N = 3; NS Non-Significant). D. Orai1 expression evaluated by Western blot 72 h after stable transfection with shOrai1 in MDA-231 shCtl and shOrai1 cells (N = 3). E. Cell adhesion assay using MTT-based technique in MDA-BrM2 cell lines (N = 3; *** *p* < 0.001). F. MTT-based cell adhesion assay of MDA-BrM2 cell line transfected with siOrai3 in presence or absence of Ca^2+^ (N = 3; *** *p* < 0.001; NS Non-Significant). G. MTT-based cell adhesion assay of MDA-BrM2 cell line transfected with ORAI3 (N = 3; *** *p* < 0.001). Figure S5: A,B. Representative fluorescence pictures (A) and quantification (B) of calpain substrate CMAC, t-BOC-Leu-Met in MDA-BrM2 cell line (siCtl n = 200; siOrai3 n = 197; N = 3; *** *p* < 0.001). C/D. Measurement of adhesion forces by means of single cell force spectroscopy in MDA-231 (C) and MDA-BrM2 (D) cell lines (MDA-231 siCtl n = 7; MDA-231 siOrai3 n = 7; MDA-BrM2 siCtl n = 7; MDA-BrM2 siOrai3 n = 7; N = 3; NS Non-Significant). Figure S6: A,B. Representative images (A) and summary of the circularity index (B) of MDA-BrM2 morphology (siCtl n = 312; siOrai3 n = 299; N = 3; *** *p* < 0.001). C. Fluorescent rhodamine phalloidin staining showing the actin architecture of siCtl and siOrai3 MDA-BrM2 cells (N = 3) (Scale bar: 10 μm). D,E. Representative images and summary of the circularity index of MDA-231 (D) and MDA-BrM2 (D) morphology transfected with CTL or ORAI3 (MDA-231 CTL n = 211; MDA-231 ORAI3 n = 228; MDA-BrM2 CTL n = 231; MDA-231 ORAI3 n = 237; N = 3; *** *p* < 0.001). Figure S7: A,B. Representative pictures (A) and summary of the circularity index (B) of MDA-BrM2 morphology (siCtl 1.8 mM n = 254; siOrai3 1.8 mM n = 257; siCtl 0.2 mM n = 199; siOrai3 0.2 mM n = 231; N = 3; * *p* < 0.05; *** *p* < 0.001; NS Non-Significant). C. Fluorescent rhodamine phalloidin staining showing the actin architecture of siCtl and siOrai3 MDA-BrM2 cells cultivated in 1.8 mM or 0.2 Ca^2+^ medium (N = 3) (Scale bar: 10 μm). Figure S8: A. Representative Western blot and quantification of FAK expression and FAK phosphorylation in MDA-BrM2 cells transfected with siCtl or siOrai3 (N = 3; *** *p* < 0.001). B. Representative Western blot of Orai3 and FAK expression after immunoprecipitation using anti-FAK antibody (N = 3; IP Immunoprecipitation). C,D. Representative fluorescence images (C) and quantification (D) of the interaction between FAK and Orai3, evaluated by PLA, in MDA-BrM2 cells (siCtl n = 401; siOrai3 n = 292; N = 3; *** *p* < 0.001). E. Representative Western blot and quantification of FAK expression and FAK phosphorylation in MDA-BrM2 cells transfected with CTL or ORAI3 (N = 3; *** *p* < 0.001). F/G. Representative Western blot and quantification of β1 integrin expression in MDA-231 (F) and MDA-BrM2 (G) cells transfected with siCtl or siOrai3 (N = 3; *** *p* < 0.001; NS Non-Significant). Scale bar: 10 μm.
