# Supplementary material for: Orai3 Calcium Channel Regulates Breast Cancer Cell Migration through Calcium-Dependent and -Independent Mechanisms
Source: Cells. 2021 Dec 10;10(12):3487. doi: 10.3390/cells10123487 (PMC8700618; doi:10.3390/cells10123487)
Supplement: Supplementary file 1 [file cells-10-03487-s001.zip › cells-1482395-supplymentary.pdf]

cells

MDPI

*Supplementary Materials*

# Orai3 Calcium Channel Regulates Breast Cancer Cell Migration through Calcium-Dependent and -Independent Mechanisms

**Mohamed Chamlali [1], Sana Kouba [1], Lise Rodat-Despoix [1], Luca Matteo Todesca [2], Zoltán Pethö [2], Albrecht Schwab [2] and Halima Ouadid-Ahidouch [1,*]**

[1] Laboratory of Cellular and Molecular Physiology, UR UPJV 4667, University of Picardie Jules Verne, 33 rue Saint Leu, 80000 Amiens France; mohamedchamlali80@hotmail.fr (M.C.); sana.kouba@u-picardie.fr (S.K.); lise.despoix@u-picardie.fr (L.R.D.); halima.ahidouch-ouadid@u-picardie.fr (H.O.A.)
[2] Institute of Physiology II, University of Münster, Robert-Koch-Str. 27b, 48149 Münster, Germany; todescal@uni-muenster.de (L.M.T.); pethoe@uni-muenster.de (Z.P.); aschwab@uni-muenster.de (A.S.)
* Correspondence: halima.ahidouch-ouadid@u-picardie.fr (H.O.A); Tel.: +33 322827646

## 1. Transient Transfections

**Table S1.** siRNA sequences.

| *siRNA* | Sequence |
|---|---|
| *siCtl* | 5′-UAGCGACUAAACACAUCAA-3′ (Eurogentec) |
| *siOrai3* | 5′-GGGUCAAGUUUGUGCCCAU-3′ (Eurogentec) |

## 2. Quantitative Real-Time PCR (qRT-PCR)

**Table S2.** Primers sequences.

| *Name* | Forward primer | Reverse primer |
|---|---|---|
| *Orai1* | 5′-AGGTGATGAGCCTCAACGAG-3′ | 5′-CTGATCATGAGCGCAAACAG-3′ |
| *Orai2* | 5′-TACCTGAGCAGGGCCAAG-3′ | 5′-GGTACTGGTACTGCGTCTCAA-3′ |
| *Orai3* | 5′-CCAAGCTCAAAGCTTCCAGCC-3′ | 5′-CAAAGAGGTGCACAGCCACCA-3′ |
| *STIM1* | 5′-CTTCAGCACAGTCCCTGTCA-3′ | 5′-TGTGGAGCTGCCTCAGTA-3′ |
| *STIM2* | 5′-AAACACAGCCATCTGCACAG-3′ | 5′-GGGAAGTGTCGTTCCTTTGA-3′ |
| *GAPDH* | 5′-AGGGGCCATCCACAGTCTTC-3′ | 5′-AGAAGGCTGGGGCTCATTTG-3′ |

## 3. Western Blot

**Table S3.** Primary antibodies, dilution and manufacturer.

| | | |
|---|---|---|
| ***Orai3*** | 1/1000 | Abcam |
| ***Orai1*** | 1/500 | Sigma |
| ***STIM1*** | 1/1000 | Cell signalling |
| ***STIM2*** | 1/1000 | Abcam |
| ***FAK*** | 1/1000 | Cell Signalling |
| ***Phospho-FAK$^{Y397}$*** | 1/1000 | Cell Signalling |
| ***GAPDH*** | 1/5000 | Abcam |

## 4. Single-Cell Force Spectroscopy

The absolute strength of breast cancer cell adhesion to a collagen I matrix was determined by means of single cell force spectroscopy using an atomic force microscop (AFM, CellHesion® 200 module, JPK, Berlin, Germany) as previously described [1]. Prior to all experiments, a tipless cantilever (ARROW-TL1, NanoWorld, Switzerland) was coated with 8µL 1 mg/mL wheat germ agglutinin (WGA, Sigma) diluted in PBS for 20 min. MDA-

231 and MDA-BrM2 cells were detached with EDTA (0,02%)/trypsin solution and resuspended in a 15 mL Falcon tube on ice. 10 µL of the cell suspension was then seeded into a 35 mm diameter glass bottom dish (Ibidi, Gräfelfing, Germany) containing 2 mL Ringer's solution (NaCl 122,5 mM, KCl 5,4 mM, $CaCl_2$ 1,2 mM, $MgCl_2$ 0,8 mM, D-glucose 5,5 mM, pH 7,4). Half of the dish surface was coated with gelatin, the other half with collagen I. The cantilever was smoothly positioned under optical control above a spherical cell. A maximum loading force of <1 nN and 2 – 10s contact time were used to firmly attach the breast cancer cell to the front part of the WGA-coated cantilever which was then lifted. The procedure of the "cell picking" was highly standardized in order to minimize variations in cell geometry and adhesion forces between the coated cantilever and the picked cell. The following parameters were used during experiments: pulling length was set to 100 µm to ensure a complete separation of the cells from the matrix; approach and retractation speed were set to 5 µm/s. The cell attached to the cantilever was lowered onto the collagen I matrix under optical control with a maximal loading force of 1 nN. After 2s or 10s of contact, the cantilever was lifted and the force required for detaching was derived from the corresponding force-distance curve. Adhesion of each cell was measured 20 times. We performed $n$ = 7 independent experiments for every condition.

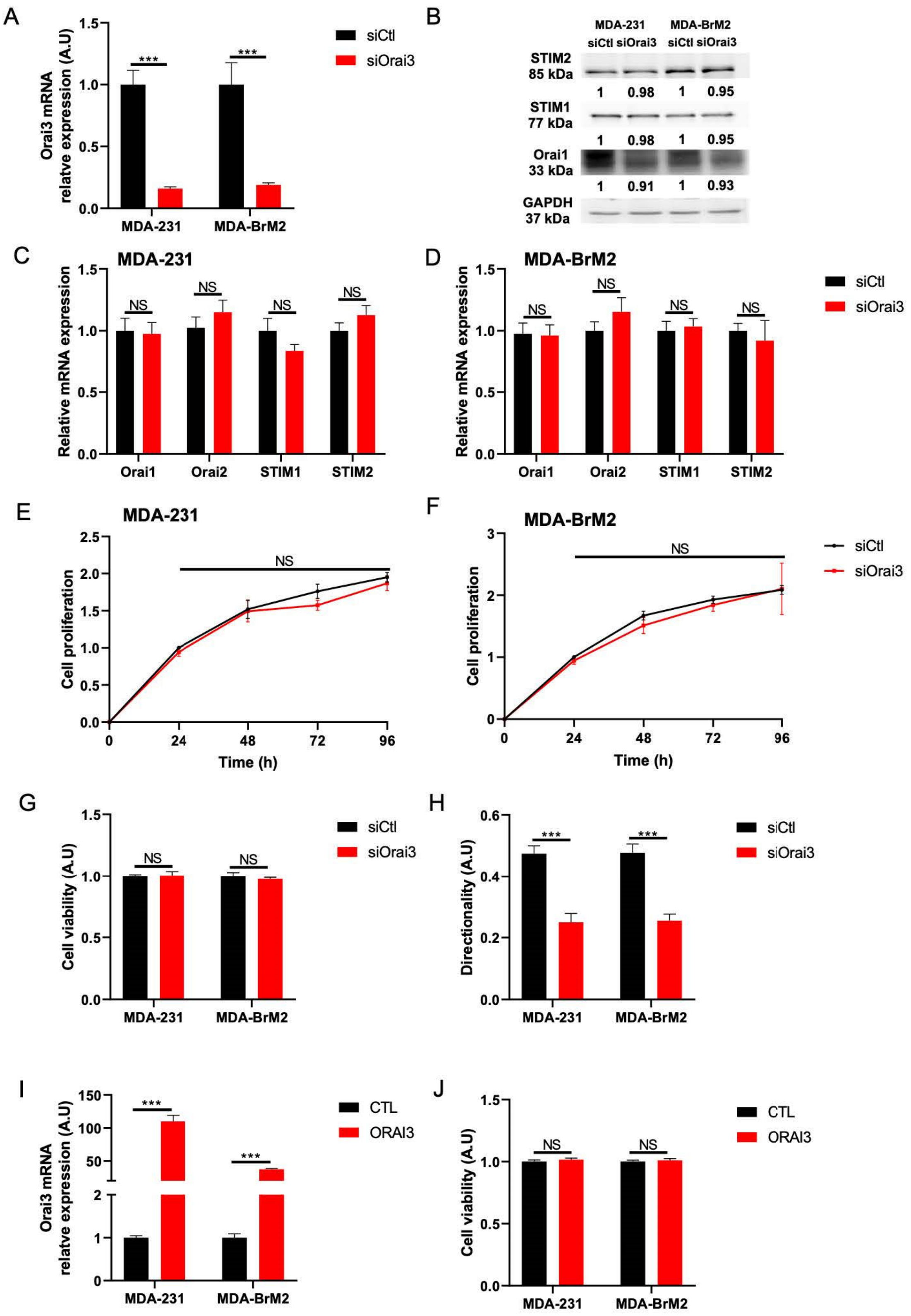


**Figure S1. A**. Orai3 expression evaluated by qPCR 72h after transfection with siOrai3 in MDA-231 and MDA-BrM2 cell lines (N = 3; *** $p < 0.001$). **B**. Orai1, STIM1 and STIM2 expression in MDA-231 and MDA-BrM2 cell lines evaluated by Western blot 72h after transfection with siOrai3 (N = 3). **C/D**. Orai1, Orai2, STIM1 and STIM2 expression in MDA-231 (C) and MDA-BrM2 (D) cell lines evaluated by qPCR 72h after transfection with siOrai3 (N = 3; NS Non Significant). **E/F**. Cell proliferation assay, using MTT-based assay, in MDA-231 (E) and MDA-BrM2 (F) cells transfected with siOrai3 (N = 3; NS Non Significant). **G.** Cell viability assay, using MTT-based assay, in MDA-231 and MDA-BrM2 cells transfected with siOrai3 (I) or ORAI3 (J) (N = 3; NS Non Significant). **H**. Cell directionality of MDA-231 and MDA-BrM2 cell lines transfected

with siCtl or siOrai3 (MDA-231 siCtl n = 49; MDA-231 siOrai3 n = 43; MDA-BrM2 siCtl n = 39; MDA-BrM2 siOrai3 n = 42; N = 3; *** p < 0.001). **I**. Orai3 overexpression in MDA-231 and MDA-BrM2 cell lines evaluated by qPCR 72h after transfection with ORAI3 (N = 3; *** p < 0.001). **J**. Cell viability assay, using MTT-based assay, in MDA-231 and MDA-BrM2 cells transfected with ORAI3 (N = 3; NS Non Significant).

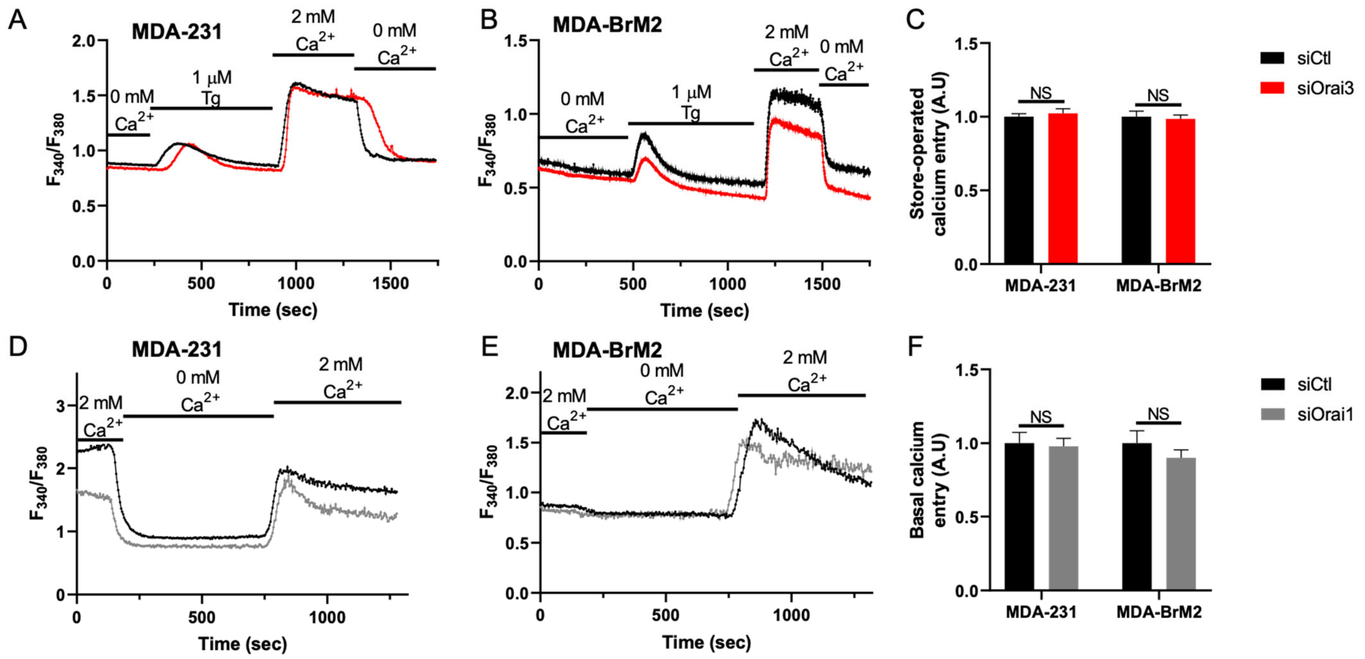


**Figure S2. A/B/C.** Traces (A/B) and quantification (C) of SOCE measured by the calculation of the $F_{340}/F_{380}$ in MDA-231 (A/C) and MDA-BrM2 (B/C) cell lines transfected with siCtl or siOrai3 (MDA-231 siCtl n = 198; MDA-231 siOrai3 n = 156; MDA-BrM2 siCtl n = 134; MDA-BrM2 siOrai3 n = 142; N = 3; *** p < 0.001). **D/E/F.** Traces (D/E) and quantification (F) of basal $Ca^{2+}$ entry measured by the calculation of the $F_{340}/F_{380}$ in MDA-231 (D/F) and MDA-BrM2 (E/F) cell lines transfected with siCtl or siOrai1 (MDA-231 siCtl n = 90; MDA-231 siOrai3 n = 88; MDA-BrM2 siCtl n = 94; MDA-BrM2 siOrai3 n = 89; N = 3; *** p < 0.001).

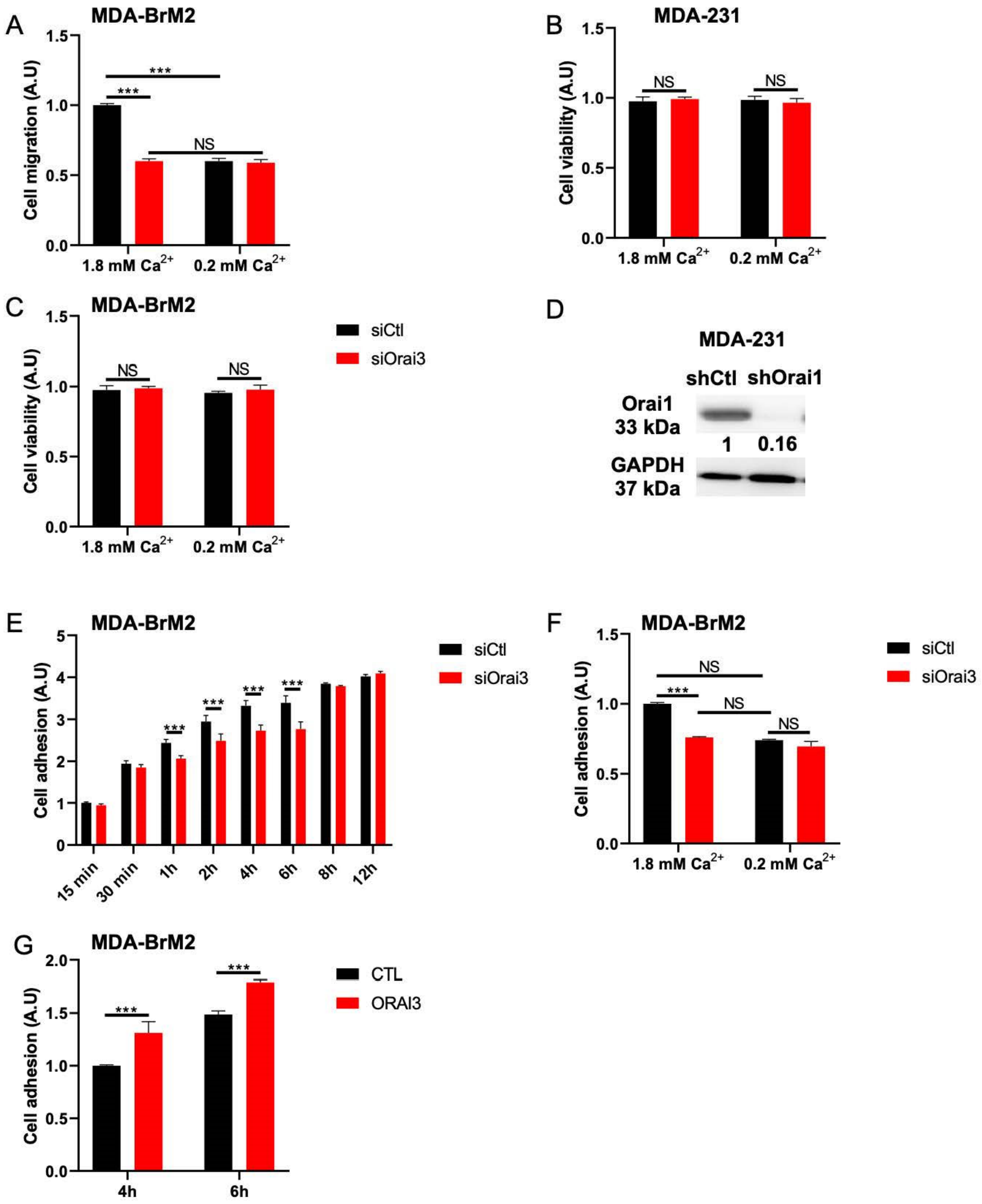


**Figure S3. A.** Migration assay using Boyden chambers with MDA-BrM2 cells transfected with siOrai3 in the presence or absence of $Ca^{2+}$ (A) (N = 3; *** $p < 0.001$; NS Non Significant). **B/C**. Cell viability assay, using MTT-based assay, in MDA-231 (B) and MDA-BrM2 (C) cells transfected with siOrai3 (N = 3; NS Non Significant). **D**. Orai1 expression evaluated by Western blot 72h after stable transfection with shOrai1 in MDA-231 shCtl and shOrai1 cells (N = 3). **E**. Cell adhesion assay using MTT-based technique in MDA-BrM2 cell lines (N = 3; *** $p < 0.001$). **F.** MTT-based cell adhesion assay of MDA-BrM2 cell line transfected with siOrai3 in presence or absence of $Ca^{2+}$ (N = 3; *** $p < 0.001$; NS Non Significant). **G.** MTT-based cell adhesion assay of MDA-BrM2 cell line transfected with ORAI3 (N = 3; *** $p < 0.001$).

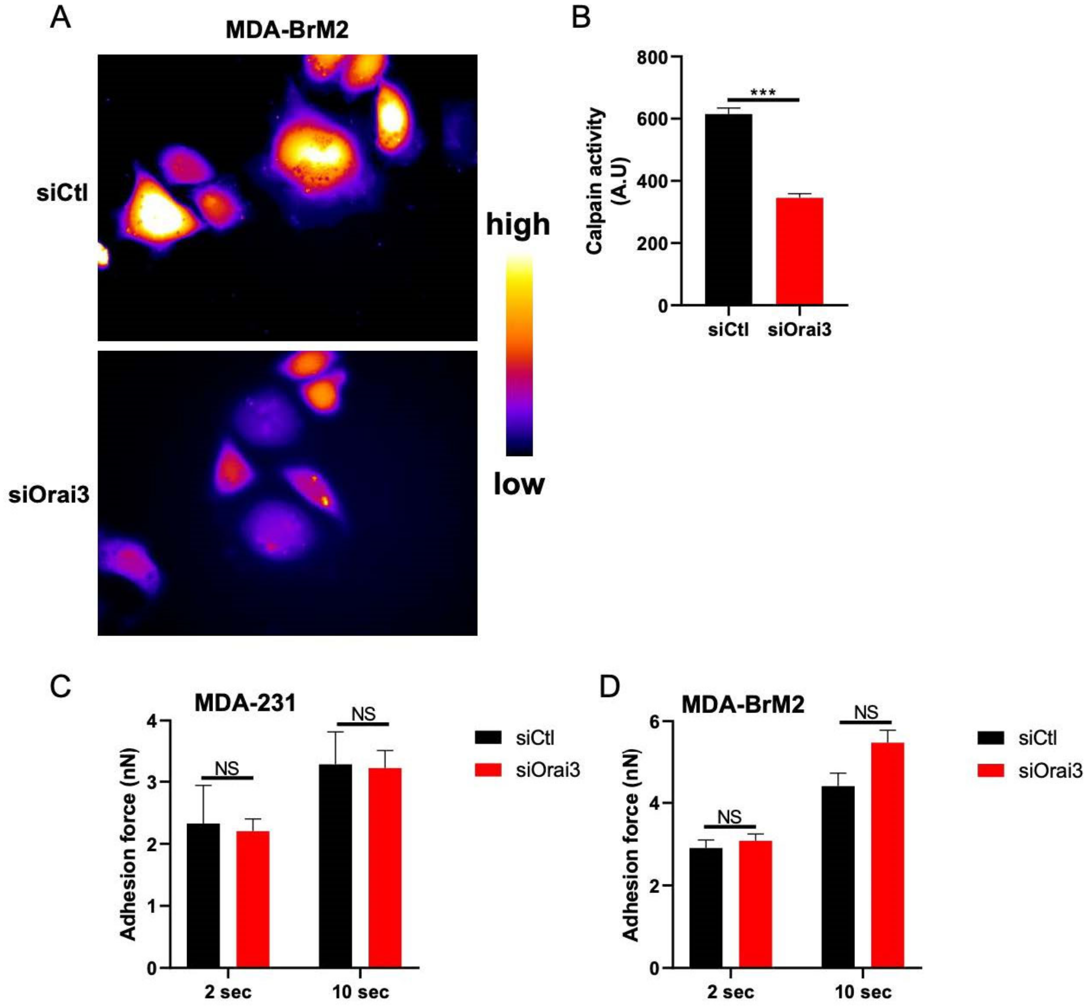


**Figure S4. A/B**. Representative fluorescence pictures (A) and quantification (B) of calpain substrate CMAC, t-BOC-Leu-Met in MDA-BrM2 cell line (siCtl n= 200; siOrai3 n= 197; N = 3; *** $p < 0.001$). **C/D**. Measurement of adhesion forces by means of single cell force spectroscopy in MDA-231 (C) and MDA-BrM2 (D) cell lines (MDA-231 siCtl n= 7; MDA-231 siOrai3 n= 7; MDA-BrM2 siCtl n = 7; MDA-BrM2 siOrai3 n = 7; N = 3; NS Non Significant).

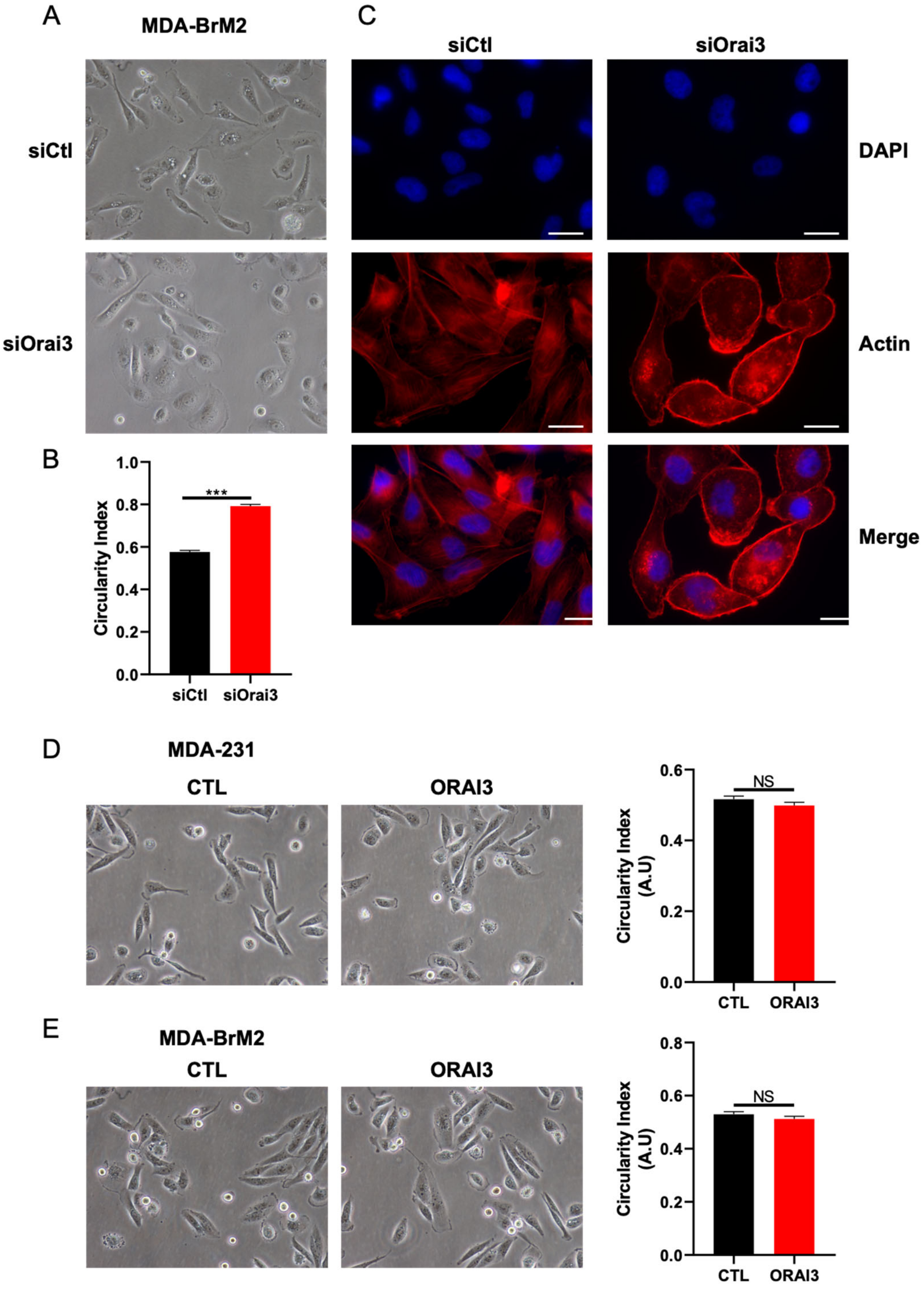


**Figure S5. A/B**. Representative images (A) and summary of the circularity index (B) of MDA-BrM2 morphology (siCtl n= 312; siOrai3 n= 299; N = 3; *** p < 0.001). **C**. Fluorescent rhodamine phalloidin staining showing the actin architecture of siCtl and siOrai3 MDA-BrM2 cells (N = 3) (Scale bar: 10 µm). **D/E**. Representative images and summary of the circularity index of MDA-231 (D) and MDA-BrM2 (D) morphology transfected with CTL or ORAI3 (MDA-231 CTL n= 211; MDA-231 ORAI3 n= 228; MDA-BrM2 CTL n= 231; MDA-231 ORAI3 n= 237; N = 3; *** p < 0.001).

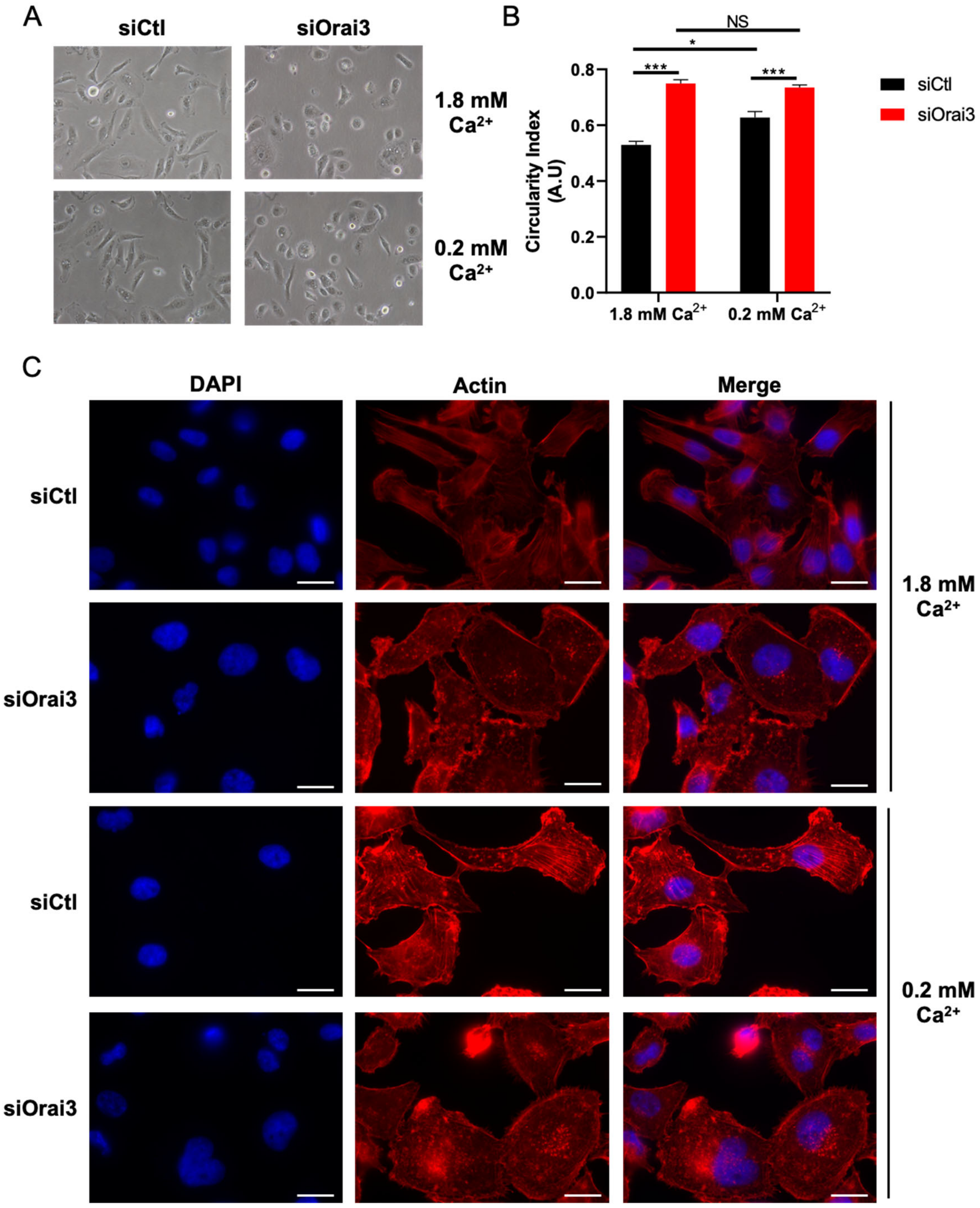


**Figure S6. A/B**. Representative pictures (A) and summary of the circularity index (B) of MDA-BrM2 morphology (siCtl 1.8 mM n= 254; siOrai3 1.8 mM n= 257; siCtl 0.2 mM n= 199; siOrai3 0.2 mM n= 231; N = 3; * $p < 0.05$; *** $p < 0.001$; NS Non Significant). **C**. Fluorescent rhodamine phalloidin staining showing the actin architecture of siCtl and siOrai3 MDA-BrM2 cells cultivated in 1.8 mM or 0.2 $Ca^{2+}$ medium (N = 3) (Scale bar: 10 µm).

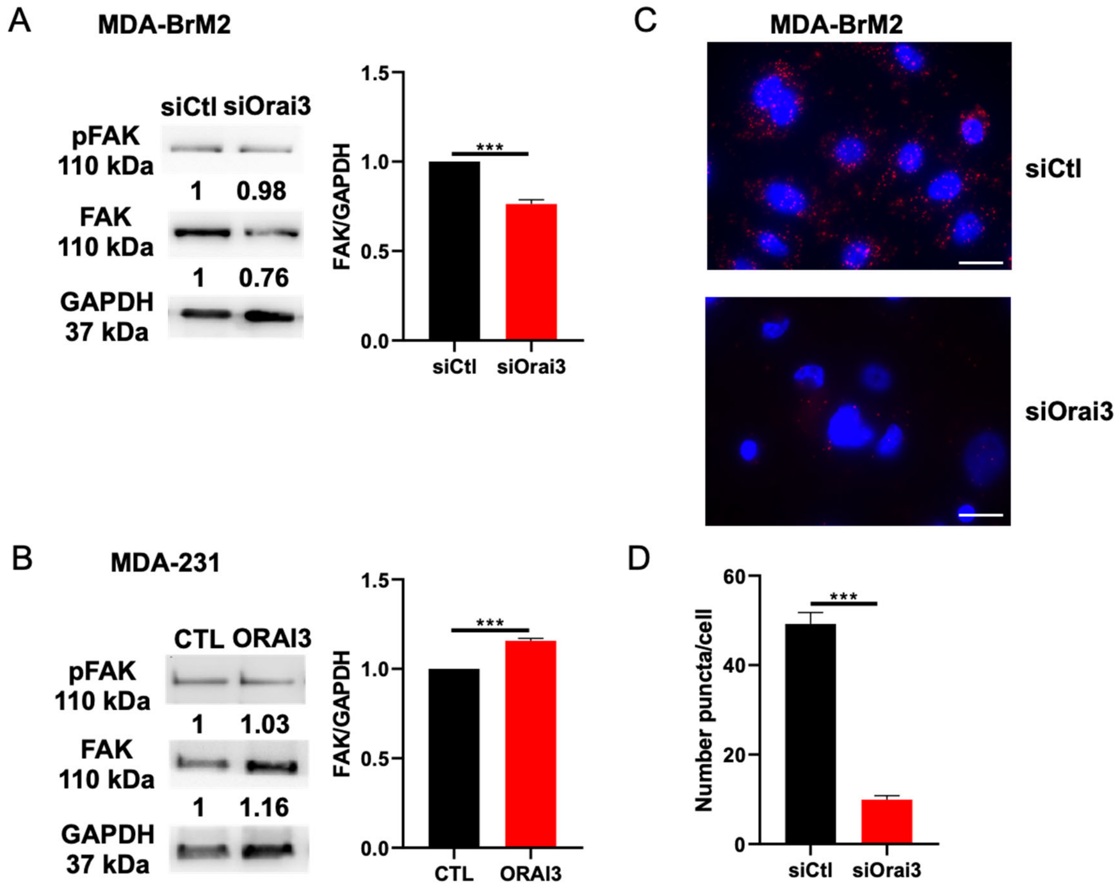


**Figure S7. A**. Representative Western blot and quantification of FAK expression and FAK phosphorylation in MDA-BrM2 cells transfected with siCtl or siOrai3 (N = 3; *** p < 0.001). **B**. Representative Western blot and quantification of FAK expression and FAK phosphorylation in MDA-BrM2 cells transfected with CTL or ORAI3 (N = 3; *** p < 0.001). **C/D**. Representative fluorescence images (C) and quantification (D) of the interaction between FAK and Orai3, evaluated by PLA, in MDA-BrM2 cells (siCtl n = 401; siOrai3 n = 292; N = 3; *** p < 0.001) (Scale bar: 10 µm).

## References

1. Bulk, E., et al., *KCa3.1 channel inhibition leads to an ICAM-1 dependent increase of cell-cell adhesion between A549 lung cancer and HMEC-1 endothelial cells.* Oncotarget, 2017. **8**(68): p. 112268-112282.
